# Trans-generational maintenance of mitochondrial DNA integrity in oocytes during early folliculogenesis

**DOI:** 10.1371/journal.pgen.1011562

**Published:** 2025-12-03

**Authors:** Qin Xie, Haibo Wu, Jiaxin Qiu, Junbo Liu, Xueyi Jiang, Huihui Wu, Qifeng Lyu, Hui Long, Wenzhi Li, Shuo Zhang, Yuxiao Zhou, Yining Gao, Aaron J. W. Hsueh, Yanping Kuang, Lun Suo

**Affiliations:** 1 Department of Assisted Reproduction, Shanghai Ninth People’s Hospital, Shanghai Jiao Tong University School of Medicine, Shanghai, China; 2 Division of Reproductive Biology, Department of Obstetrics and Gynecology, Stanford University School of Medicine, Stanford, California, United States of America; Instituto Leloir, ARGENTINA

## Abstract

Mutations in mitochondrial DNA (mtDNA) can lead to mitochondrial and cellular dysfunction. However, recent studies suggest that purifying selection acts against mutant mtDNAs during transgenerational transmission. We investigated the mtDNA dynamics during ovarian follicle development. Using base-editing, we generated mice harboring a 3177 G > A mutation corresponding to the human Leber hereditary optic neuropathy (LHON)-related mtDNA mutation and confirmed a transgenerational reduction of the mutant mtDNA. Utilizing a mouse follicle culture system in which pathogenic mtDNA mutations were introduced *in vitro,* followed by mtDNA sequencing and digital PCR, we found that the germline heteroplasmy shift during early folliculogenesis was driven by a decrease in mutant mtDNA along with compensatory replication of wild-type mtDNA. In contrast, synonymous mtDNA mutations did not affect mtDNA dynamics. These findings demonstrate that mice can eliminate certain pathogenic mtDNA mutations in the germline during early folliculogenesis, thus advancing our understanding of mtDNA purifying selection during oogenesis. Furthermore, our use of mtDNA editing in in vitro-cultured follicles provides a novel approach to create and monitor mitochondrial DNA mutations.

## Author summary

Mitochondria are energy generators inside our cells, and they carry their own DNA (mtDNA). Due to inadequate DNA repair mechanisms in the mitochondria, mitochondrial genome contains more mutations than that of the nuclear genome, leading to serious diseases, especially in organs that requiring abundant energy supply. However, the body could reduce the risk of passing these mutations to the next generation through purifying selection. Our work explored how this process works during ovarian follicle development in mice. These findings help explain how the body protects future generations from mitochondrial diseases.

## Introduction

The mitochondrial genome (mtDNA) is a circular, double-stranded DNA located within the mitochondrial matrix and encodes 37 genes critical for oxidative phosphorylation [1]. There are thousands of copies of mtDNA in each somatic cell, but more than 100,000 copies in a mature oocyte. Due to replication errors, inadequate proofreading and repair mechanisms, and a strict maternal inheritance pattern [2], mtDNA, unlike the nuclear genome, is highly susceptible to the accumulation of pathogenic mutations [3,4]. When the proportion of mutations (heteroplasmy) in critical regions of mtDNA exceeds a certain threshold, it can lead to severe diseases such as Leber hereditary optic neuropathy (LHON), mitochondrial encephalomyopathy, lactic acidosis and stroke-like episodes (MELAS) [5,6].

Recent studies suggest that mutant mtDNAs are reduced during transgenerational transmission. The elimination of pathogenic mutations across generations, known as germline purifying selection, was first observed in PolgA mutant mice [7] and in mice with a mutation in *ND6* [8]. This phenomenon has also been found in *Drosophila melanogaste*r [9–12] and nematodes [13]. Similarly, mother-offspring pairs in humans have shown a reduction of pathogenic mutations across generations, based on large-scale observational data [14–17].

Purifying selection is of paramount importance for the adaptation and propagation of populations while avoiding extinction, but the mechanisms remain poorly understood. To be transmitted to offspring, mtDNA must first segregate into primordial germ cells (PGCs) and then replicate during prolonged folliculogenesis [18,19]. However, the difficulties in culturing germ cells *in vitro* and manipulating mtDNA mutations have limited progress in this area.

Here, we employed a novel mtDNA editing tool-DdCBE [20] to introduce pathogenic mutations associated with human diseases in mice and in follicle culture models [21–26].

Leber hereditary optic neuropathy (LHON) is one of the most common mitochondrial diseases in humans. The m. 3733G > A mutation in *ND1* has been reported as a LHON-associated mutation, which leads to degeneration of retinal ganglion cells and severe bilateral visual loss in patients [27–30]. Human m. 3733G > A and its corresponding site m. 3177G > A in mice convert glutamic acid into lysine. By generating both an *in vivo* mouse model and an *in vitro* follicle model, we investigated the dynamics of mtDNA purifying selection during folliculogenesis.

In this work, we first provided evidence that pathogenic, but not synonymous, mtDNA mutations can be eliminated during folliculogenesis. Second, we observed that selection against mtDNA mutations occurs at the organelle level via two pathways: compensatory replication of wild-type mtDNA copies and elimination of mitochondria containing high levels of mutant mtDNA, likely through mitophagy. Moreover, the experimental platform developed in this work offers a tool to investigate the molecular mechanism of purifying selection within follicles.

## Materials and methods

### Ethics statement

All experimental procedures were approved by the Institutional Animal Care or Research Ethics Committee at Shanghai Jiao Tong University School of Medicine.

### Mice

C57/B6J mice used were from Beijing Vital River Laboratory Animal Technology Co., Ltd. and maintained at 23°C under a 12h:12h light-dark cycle.

### Plasmids generation

The TALE-based DdCBEs vectors are composed of mitochondrial localization sequence (MTS), N-terminal and C-terminal domains, one of the four split DddA halves, and UGI-coding sequences [20]. The TALE RVDs were first assembled using the Golden-TALE system and inserted into the DddAtox vector via Esp3I digestion. S1 Table provides detailed information of the DddAtox construct and the binding arrays of each DdCBE element.

### In vitro Transcription

TALE-based DdCBE plasmids with T7 promoters were linearized with NotI (NEB) endonuclease and purified using 1.2% agarose gel electrophoresis. Following the manufacturer’s manual, the purified product was used as a template for in vitro transcription (IVT) using the mMESSAGE T7 ULTRA kit from Life Technologies. To prepare DdCBE mRNA for injection into germ cells, both halves were further purified using the MEGAclear kit from Life Technologies.

### Construction of mtDNA mutant animals

To construct an animal model with the m.3177 G > A mutation, six-week-old female C57BL/6 mice were super-ovulated for zygote collection via intraperitoneal injections of 10 IU of PMSG, followed by 10 IU of hCG (Ningbo Hormone Products Co., China) 48 hours later. Male and female mice were then allowed to mate. Twenty hours after hCG administration, fertilized eggs with two pronuclei were collected from oviducts and placed in M2 medium. The cumulus mass was removed using 70μg/mL hyaluronidase prior to microinjection. After one hour of recovery, pronuclear embryos were injected with10pL mixture of 300ng/ul forward and 300ng/ul reverse TALEs based DdCBE-3177. The zygotes were then cultured in KSOM medium at 37°C with 5% CO_2_ in air under mineral oil. To generate the mouse model, two-cell stage embryos were transferred into the fallopian tubes of pseudo-pregnant recipient mice.

### Follicle culture in vitro

A detailed method has been described previously22–26 [31]. Briefly, 12-day-old B6D2F1 females were used to collect secondary follicles with diameters between 105 and 125μm. Isolated follicles with an intact structure were chosen for microinjection and in vitro culture. 300ng/ul left halves and 300ng/ul right havles of DdCBE were equally mixed, and 10pL of the mixture was microinjected into the cytoplasm of oocytes from secondary follicles after a one-hour recovery period.

Following microinjection, the follicles underwent a 25-minute collagenase treatment to dissolve the follicular wall. Follicles were then cultured in α-MEM supplemented with 5% fetal bovine serum, 1% GlutaMax (Gibco), 2% PVP, 0.1% Penicillin-Streptomycin (Gibco), 150M 2-O-alpha-d-glucopyranosyl-l-ascorbic acid (AA2G, Tokyo Chemical Industry), and 100mU/mL follicle-stimulating hormone (FSH, Gonal-F). The cultures were maintained at 37°C, 100% humidity, and 5% CO_2_ in air, with half of the medium replaced every other day.

### DNA extraction

Genomic DNA from mouse tissues was extracted using the DNeasy Blood & Tissue kit (Qiagen). To extract DNA from a single oocyte, follicles were digested using a solution containing 0.5% trypsin (Gibco), 0.1% collagenase Type I (Worthington), 0.1% DNase I (Sigma), and 1mM EDTA (Solarbio) for 10 minutes. Oocytes were then stripped of surrounding granulosa cells and rinsed three times in a solution containing 0.4% BSA. Subsequently, they were immersed in 5μl of QuickExtract DNA Extraction Solution for digestion.

### Digital PCR

The primers and probes were purchased from IDT, as listed in S2 Table. A 5μl sample containing a single oocyte was added with 10μl of DNase-free ddH2O. Subsequently, 2μl of this mixture was utilized as a template for PCR amplification. The reaction droplets were generated using the droplet generator of the droplet digital PCR Instrument from a 20μl PCR reaction mix. The droplets were then amplified on the Bio-Rad T100 PCR instrument using the following cycling conditions: initial denaturation step at 95°C for 10 minutes, followed by 40 cycles of denaturation at 94°C for 30 seconds, annealing at 60°C for 1 minute, and extension at 72°C for 1 minute. The final extension step was performed at 98°C for 10 minutes. After amplification, fluorescence signals emitted by FAM (representing wild-type copies) and HEX (representing mutant copies) in each droplet were detected using the QX200 Droplet Reader and analyzed with QuantaSoft software.

### Target sequencing

A total of 100ng of genomic DNA obtained from mice tail or 5ul single oocyte lysates was utilized for the initial PCR amplification. Phanta Flash DNA Polymerase (Vazyme) and primers with incorporated barcodes and Illumina adapters (S2 Table) were used to amplify the desired target sequences. One microliter of the PCR product was then used for a second-round PCR with index primers from Vazyme. After completion of the second-round PCR, samples with distinct barcodes and indexes were pooled and purified by gel extraction using the QIAquick Gel Extraction Kit (Qiagen). The purified products were then quantified using the Qubit ssDNA HS Assay Kit (Thermo Fisher Scientific).

Deep sequencing was performed on the Illumina NovaSeq 6000 platform. Sequencing data were quality-checked using fastp (v0.23.2). The sequencing reads were demultiplexed with fastq-multx (v1.4.1) using the barcoded PCR primers. Mutation rates at on-target sites were subsequently determined by analyzing the output files from batch processing with CRISPResso2 (v2.0.32). Statistical analysis was conducted using custom scripts developed in R (v4.2.1).

### Whole mtDNA sequencing

Whole mitochondrial DNA (mtDNA) was amplified using long-range PCR, which involved the amplification of two overlapping 8kb fragments. The primer sequences used are listed in S2 Table. PCR products were purified using the QIAquick Gel Extraction Kit (Qiagen) and subsequently utilized as input for library construction using the DNA Library Prep Kit V2 for Illumina (Vazyme). Before deep sequencing, the libraries were purified using DNA clean beads and quantified with the Qubit ssDNA HS Assay Kit (Thermo Fisher Scientific).

For the analysis of next-generation sequencing data from whole mitochondrial genome sequencing, the qualified reads were first aligned to the mitochondrial reference genome (mm10) using BWA (v0.7.12) with the mem–M option. BAM files were then generated using SAMtools (v.1.9). Positions with conversion rates ≥ 1% among all cytosines and guanines in the mitochondrial genome were identified using the REDItoolDenovo.py script from REDItools (v.1.2.1).

### Whole genome off-target detection

To detect potential TALEN off-target effects, we performed whole-genome sequencing (WGS) analysis. Briefly, genomic DNA was extracted and sonicated to generate 300–500 bp fragments. The fragmented DNA was then equipped with adaptors, PCR-amplified, and sequenced on the MGI platform.

Raw sequencing data underwent quality control processing using Fastp [32] for filtering low-quality reads. High-quality reads were subsequently aligned to the hg38 reference genome using BWA [33]. Duplicate reads were removed with Picard tools, followed by base quality score recalibration using GATK (v4.6.1). Genome-wide variant calling was performed using GATK’s HaplotypeCaller, with SNP identification achieved through the SelectVariants function (select-type SNP). VariantFiltration was applied to remove low-quality SNPs. Mitochondrial DNA variants were separately analyzed using Mutect2 with --mitochondria-mode parameter.

Potential off-target sites were predicted using TALENoffer [34], generating a list of top 10,000 candidate loci. Intersection analysis between these predicted sites and the identified genomic/mitochondrial SNPs revealed only one overlapping variant, indicating precise genome editing with minimal off-target effects.

### Transmission electron microscope observation

Secondary follicles were isolated and cultured in vitro. Oocytes were dissected three days post-injection and prepared for transmission electron microscopy (TEM). Specifically, oocytes were immersed in a solution consisting of 2.5% glutaraldehyde and 2% paraform for 2 hours at 4°C. Following fixation, the specimens were washed twice with phosphate buffer and then post-fixed using 1% osmium tetroxide.

Dehydration was carried out through sequential immersions in increasing concentrations of ethanol (30%, 50%, 70%, 80%, 95%, and 100%) for approximately 10 minutes at each step. This was followed by two changes of 100% propylene oxide (P.O.) for 15 minutes each. The specimens were then placed in a mixture of P.O. and embedding resin (Epon 812) in gradually increasing resin concentrations and finally immersed in pure embedding resin (Epon 812) for 6 hours at 37°C.

The specimens were encapsulated in embedding medium and subjected to heating at 60°C for 48 hours. Subsequent steps involved sectioning the samples to 70–90nm using a diamond knife (Leica EM UC7), followed by staining with uranyl acetate and lead citrate before observation under a transmission electron microscope (HITACHI H-7650 or FEI Talos 120).

### Quantification and statistical analysis

For the method of mapping the density curve, we first generated the density curve of the observed heteroplasmy shift. Then we generated 1000 times the sample size random value based on the Kimura distribution and drew the density curve. For data analysis, the normality of the data was evaluated through the Shapiro-Wilk test. If the data satisfied the assumptions of normality, we employed an unpaired Student’s t-test to examine the differences between groups. If the data failed to satisfy the assumptions of normality or equal variances, we utilized the Mann-Whitney U test. Statistical analysis was performed by GraphPad Prism 9 and P < 0.05 will be considered as significant. The statistical details could be found in each Fig legend.

## Results

### The pathogenic m.3177G > A mutation was eliminated in mice during germline transmission

**Fig 1:**
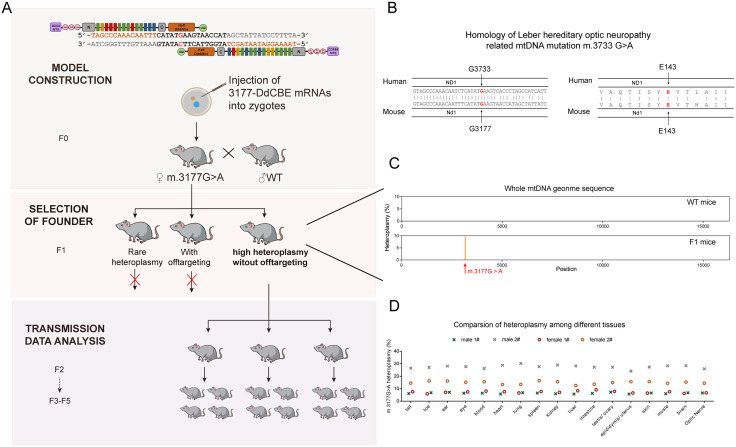
Generation of m.3177 G > A mutant mice. **(A)** The experimental workflow for generating the m.3177 G > A mice by injecting base editing tool-DdCBE-3177 mRNAs- into zygotes and selection of high heteroplasmy lines without off-targeting. **(B)** Homology of the human Leber hereditary optic neuropathy mtDNA mutation m.3733 G > A and corresponding mouse mutant selected. **(C)** Lack of off-targeting in the mtDNA genome in the F1 m.3177 G > A mice with the heteroplasmy of 6.72%. A wildtype (WT) mouse was used as the control. **(D)** The heteroplasmy among different tissues in m.3177 G > A mice. F represent generation and m represent mice age in months.

**Fig 2:**
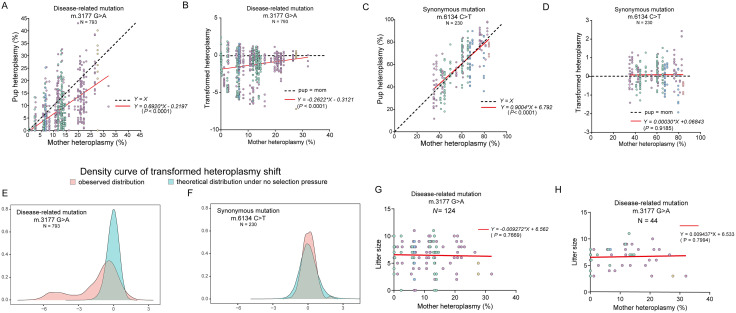
Germline transmission of mtDNA m.3177 G > A mutation in mice. **(A)** Transmission of heteroplasmy from 44 mothers to 793 pups in disease-related m.3177 G > A mutant mice. **(B)** The transformed heteroplasmy shift of disease-related m.3177 G > A mutant mice shown in Fig 2A. **(C)** Transmission of heteroplasmy from 20 mothers to 230 pups of a synonymous mutation in m.6134 C > T mice. **(D)** The transformed heteroplasmy shift of synonymous mutation with m.6134 C > T mice shown in Fig 2C. **(E-F)** Density curve of transformed heteroplasmy shift of mother-pup transmission of **m.** 3177G > A mutation **(E)** and **m.** 6134T > C mutation **(F)**. **(G)** The litter sizes of 124 deliveries in **m.** 3177G > A mice. **(H)** The litter size at 44 first deliveries of m.3177G > A mice. The transformed heteroplasmy shift was calculated as ln(*h*(1-*h*_*0*_)/*h*_*0*_(1-*h*)). The variable “*h”* is the pup’s heteroplasmy, and “*h*_*0*_” represents the mother’s heteroplasmy. The red line represents the fitted curve while the dashed line indicates unbiased transmission. For panels A-F, orange, blue, green, purple, and yellow dots refer to F1, F2, F3, F4, and F5 generations, respectively.

To explore the germline transmission of mutant mtDNA, we first constructed a mouse model with the m.3177 G > A mutation (Fig 1A and 1B) using the recently developed mtDNA editing tool DdCBE [20]. Detailed information of the DdCBE elements is provided in S1 Table. *In vitro* transcribed 3177-DdCBE mRNA was purified and microinjected into zygotes to generate F0 mice. These F0 females were then backcrossed with wild-type (WT) males. As the DdCBE-based mtDNA editing system may cause off-target edits in mtDNA, we screened all F1 mice by Whole mtDNA sequencing to minimize potential effects of off-targeting on the analysis of germline transmission. To avoid the contamination of nuclear mitochondrial DNA segments, the whole mtDNA was amplified as two overlapping 8-kb fragments by long-range PCR, and purified using gel extraction prior library preparation. A 1% threshold was used to identify off-target events. One female carrying 6.72% m.3177 G > A mutation without detectable off-target mutations in mtDNA (Fig 1A and 1C) was selected as the founder of the m.3177 G > A mouse model for this study (Fig 1A). We also performed whole-genome sequencing (WGS) analysis in this founder mouse. The potential off-target sites in the nuclear genome were predicted using TALENoffer [34], generating a list of the top 10,000 candidate loci. Intersection analysis between these predicted sites and the identified genomic/mitochondrial SNPs revealed no nuclear off-targeting (The raw sequencing data have been deposited into the NCBI’s Sequence Read Archive (Accession No. PRJNA1059054)), indicating a clean nuclear background of founder mice. We mated this female founder with WT males to start a colony and all females in this study were all mated with WT males. We then sequenced heteroplasmy levels across 17 different tissues from 20 mice spanning F1 to F5 generations. Results indicated stable heteroplasmy, with an average coefficient of variation of 8.35% (Fig 1D). Therefore, tail heteroplasmy was considered representative of whole-body heteroplasmy in subsequent analyses. To observe the purifying selection against the mtDNA mutation, we mated females carrying m. 3177 G > A mutation with WT males from the F1 to F5 generations. A total of 44 mother and their 793 pups were analyzed. As expected, the pups exhibited a wide range of heteroplasmy levels, likely due to the mtDNA bottleneck effect [18,35]. In the F1 and F2 generations, we mated all females we obtained without any artificial selection. The average heteroplasmy in F2 is 5.82%, slightly lower than the 6.72% observed in the F1 founder, while the highest heteroplasmy in F2 pups achieved was 25.2%. In the F3 generation, the average heteroplasmy further decreased to 4.79%, with a maximum of 32.01%. For F3 and F4 females, we selectively bred females with high heteroplasmy. In the F4 and F5 generation, average heteroplasmy is 8.53% and 23.40%, respectively, with a maximum values of 43.02% and 40.14%. Comprehensive analysis of heteroplasmy in mother-pup pairs revealed strong negative selection against the m.3177 G > A mutation, as evidenced by a regression slope of 0.69 on the mother-offspring fitness curve (*P* < 0.0001) (Fig 2A). To analyze the relative heteroplasmy ratio of pups as compared to the mother, we transformed the heteroplasmy by a previous reported method [36] ln(h(1-h0)/h0(1-h)), where “h” is the pup’s heteroplasmy, and “h0” represents the mother’s heteroplasmy (Fig 2B). Both analyses consistently showed that the average heteroplasmy in pups was lower than in their mothers, suggesting purifying selection against the pathogenic mutation during transgenerational transmission. We also leveraged a second mouse model with a synonymous mutation (m.6134 C > T) which arose due to DdCBE off-targeting. In contrast to the pathogenic mutation, this synonymous mutation exhibited unbiased transmission (Fig 2C-2D), indicating no purifying selection. To make the different ranges of data comparable between pathogenic and synonymous mutations, we compared the observed distributions of heteroplasmy with theoretical distributions under neutral selection predicted by the Kimura model [37]. The distribution of the pathogenic m.3177G > A mutation exhibited an obvious left skew, while the synonymous m.6134C > T mutation followed a nearly neutral distribution (Fig 2E-2F), supporting the presence of negative selection against the pathogenic variant.

To test whether the decline in heteroplasmy was derived from embryonic lethality in pups with high heteroplasmy, we analyzed the correlation between litter size and mother’s heteroplasmy ratios in m.3177 G > A mutant mice. No correlation was observed (Fig 2G, P = 0.7669). To avoid the confounding effect of maternal age on litter size, we performed a subgroup analysis using only the first litter from each mother, which yielded similar results (Fig 2H, P > 0.05).

### Elimination of the mtDNA mutation with m.3177G > A occurs in early folliculogenesis *in vivo*

**Fig 3:**
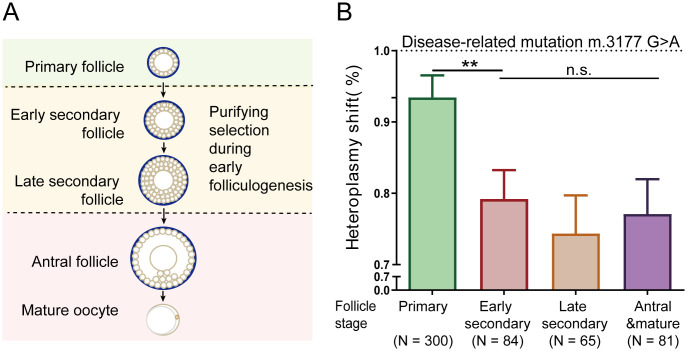
mtDNA dynamics in oocytes carrying the m. 3177 G > A mutation. **(A)** The diagram of follicle development. **(B)** The transformed heteroplasmy shift of mtDNA in oocytes at different follicle stages in m.3177 G > A mice showed a drop in heteroplasmy during the transition from primary to secondary follicle development. The heteroplasmy shift was calculated as *h*(1-*h*_*0*_)/*h*_*0*_(1-*h*). *h* is the oocyte heteroplasmy, and *h*_*0*_ is the mother’s heteroplasmy. Significance was calculated with *Mann-Whitney U* test (** *P* < 0.01).

The decreased mutation load observed in offspring without affecting litter sizes implied that selection pressure may act before ovulation, consistent with previous studies [8,38–40]. Before maturation for fertilization, oocytes within primordial follicles pass through primary, early secondary, late secondary, and antral stages (Fig 3A). A previous study reported no selection against the m.3875delC in primordial germ cells and P3.5 oocytes (follicle starting to assemble), indicating selection occurs after follicle formation [38]. In contrast, another study found an increase in the m.5024 C > T mutation in antral follicles [36]. To determine the stage at which purifying selection occurs during folliculogenesis, we isolated follicles at different developmental stages from the ovaries of four mice carrying the m.3177 G > A mutation and then sequenced the mtDNAs of their oocytes. To combine the data of mice with various levels of heteroplasmy, we normalized the absolute heteroplasmy values into heteroplasmy shift values defined as *h*(1-*h*_*0*_)/*h*_*0*_(1-*h*) following a previous reported method [36], where *h* is the oocyte’s heteroplasmy, and *h*_*0*_ is the mother’s heteroplasmy. In primary follicles, heteroplasmy showed a slight decrease from 1 to 0.93 (Fig 3B). As folliculogenesis progressed, heteroplasmy significantly declined to 0.79 at the early secondary follicle stage and further to 0.74 at the late secondary stage, levels comparable to those found in offspring (0.70) (Fig 3B). These results indicate that a substantial reduction in heteroplasmy mainly occurs during early folliculogenesis.

Consistent with a previous study reporting an age-related selection effect on the m.2820G > A mutation [35], we also observed that advanced maternal age enhanced the purifying selection of the pathogenic mtDNA mutation in a germline-specific manner (S1A-S1C Fig). The heteroplasmy shift was calculated as h(1-h0)/h0(1-h). h is the pup’s heteroplasmy, and h0 is the mother’s heteroplasmy. When the effect of purifying selection is enhanced, a lower heteroplasmy is expected to be observed. By analyzing 793 mother-pup pairs, we found that the magnitude of heteroplasmy shift declined with increasing maternal age. When we isolated 1792 oocytes from 38 mouse ovaries at different ages, an obviously negative correlation between heteroplasmy shift and maternal age was found. However, analysis of various somatic tissues from m.3177 G > A mice showed no significant correlation between maternal age and heteroplasmy shift in somatic cells.

### Follicular elimination of the mtDNA mutations in a functionally dependent manner *in vitro*

**Fig 4:**
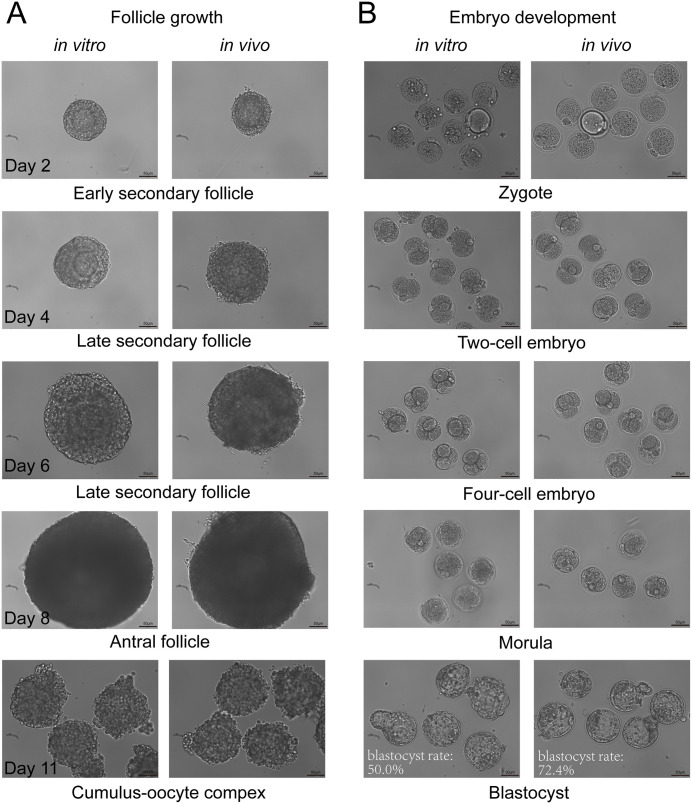
The *in vitro* development of secondary follicles. **(A)** Comparison of the morphology of secondary follicles derived from *in vivo* and *in vitro* models. **(B)** Comparison of the morphology of embryos derived from secondary follicles derived *in vivo* and *in vitro*.

To further investigate the dynamics of mitochondrial mutations during folliculogenesis, we generated oocytes carrying different types of mutations by isolating and culturing early secondary follicles from WT mice using a recently reported culture system [21–26]. The follicles cultured *in vitro* exhibited morphology similar to those grown *in vivo*, and 50% of them could reach the blastocyst stage [21,22], indicating the developmental potential of *in vitro* cultured follicles (Fig 4).

**Fig 5:**
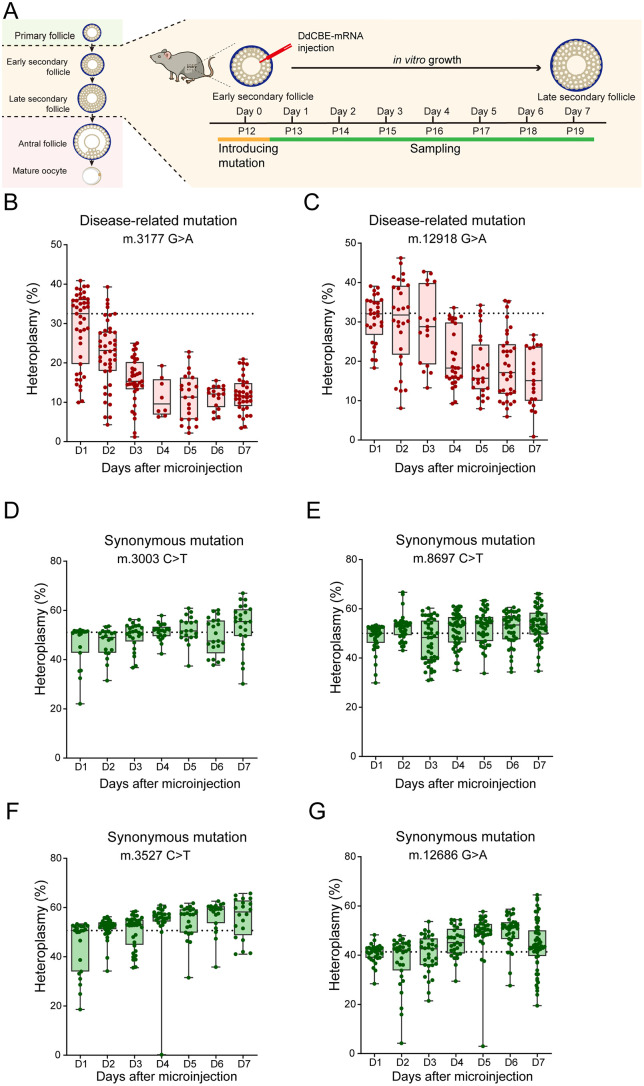
Dynamic of mtDNA disease-related and synonymous mutation rates during early folliculogenesis in *vitro.* ***(A)** In vitro* culture and microinjection of DdCBE-mRNAs for base-editing and sampling at different times during secondary follicle development. P12 to 19 denote corresponding to postnatal days *in vivo*. **(B-C)** Dynamics of disease-related mtDNA heteroplasmy for m.3177 G > A **(B)** and m.12918 G > A **(C)** mutations during secondary follicle development *in vitro*. **(D-G)** Dynamics of synonymous mtDNA heteroplasmy for m.3003 C > T **(D)**, m.8697 C > T **(E)**, m.3527 C > T **(F)**, and m.12686 G > A **(G)** during secondary follicle development *in vitro*. Data were presented from Min to Max with Median. The black dotted line represents the median heteroplasmy of Day1. Data was derived from 3 independent replicate experiments.

To observe the dynamics of the absolute heteroplasmy level during folliculogenesis, equal volumes of DdCBE mRNA were injected into oocytes to introduce similar starting mutation loads. We first injected the DdCBE-3177 mRNA into oocytes of early secondary follicles on the day of isolation (Day 0) and sampled them continuously for seven days (Fig 5A). One day after microinjection, DdCBE-3177 introduced approximately 30% mutations, followed by a sharp decline by day 4 of culture in three independent experiments (Fig 5B). We also generated another disease-related mutation, m.12918 G > A, corresponding to the human m.13513 G > A mutation causing Leigh Syndrome and MELAS [41,42]. A similar decline in heteroplasmy was observed during culture (Fig 5C). Notably, the oocyte loss rate remained below 5% across all replicates. To exclude the potential effect of off-targetting caused by DdCBE, we performed whole mtDNA sequencing of oocytes carrying these two pathogenic mutations. As shown in S2 Fig, no significant off-targetting was observed.

To determine whether purifying selection was directed against base substitutions per se or against dysfunctional mitochondria caused by pathogenic mutations, we introduced four synonymous mutations (m.3003 C > T, m.8697 C > T, m.3527 C > T, and m.12686 G > A) that altered mtDNA bases without affecting encoded proteins. in contrast to the disease-related mutations, no purifying selection was observed for these synonymous mutations during folliculogenesis (Fig 5D-5G). These findings suggest that the selection pressure likely arises from functional impairments of mitochondria caused by pathogenic mutations.

### Heteroplasmy decrease of the m.3177G > A mutation in oocytes was induced by eliminating mutant copies together with compensatory replication of wildtype variants

**Fig 6:**
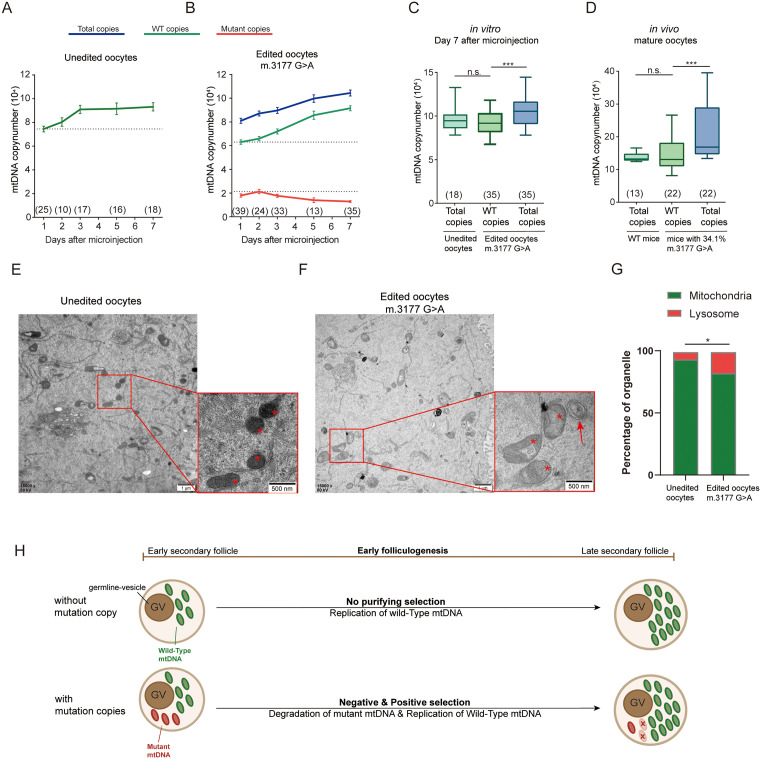
Dynamic of mtDNA copy numbers changes during early folliculogenesis. **(A)** The dynamic of mtDNA copy numbers derived from digital DNA PCR in unedited oocytes during secondary follicle development *in vitro*. The green line represents wild-type (total) copies. **(B)** The dynamic of mtDNA copy numbers in m.3177 G > A edited oocytes during *in vitro culture*. The blue line represents the total copies. The green line represents wild-type copies. The red line represents mutant copies. The error bars represent the standard error of the mean (SEM). Data was derived from 3 independent replicate experiments. **(C)** Comparison of mtDNA copy numbers in edited oocytes and unedited oocytes on Day 7 after microinjection. Significance was based on two-tailed *Student’s t-test* (*** p < 0.001). The data are presented by box plots (maximum-median-minimum). **(D)** Comparison of mtDNA copy numbers in mature oocytes from m.3177G > A and wildtype (WT) mice. Significance was calculated based on the *Mann-Whitney U* test (*** p < 0.001). The data are presented by box plots (maximum-median-minimum). **(E)** Transmission electron microscope images of mitochondria in unedited oocytes. The red asterisk points indicate the mitochondria. **(F)** Transmission electron microscope images of mitochondria in m.3177 G > A edited oocytes. The red asterisk points indicate the swollen mitochondria and the red arrow indicate the lysosome. **(G)** Comparison of the ratio of mitochondria and lysosome of unedited (8 independent slides) and edited (13 independent slides) oocytes from transmission electron microscope images. Significance was based on Chi-squared *test* (*p < 0.05). **(H)** Diagram showing dynamics of mtDNA copy number changes during early folliculogenesis. During early folliculogenesis, unedited follicles showed replication of wildtype (WT) copies while edited follicles showed simultaneously negative selection by degradation of mutant copies and compensatory replication of WT copies.

It is unclear whether decreases in heteroplasmy result from the elimination of mutant copies, compensatory upregulation of WT copies, or both [4,43,44]. To investigate the molecular dynamics underlying selection, we first employed digital PCR on oocytes from DdCBE-injected follicles to quantify the copy numbers of both WT and mutant mtDNA in individual oocytes. We examined the dynamics of mtDNA copy numbers in unedited oocytes over seven days of culture and found a rapid increase to approximately 100,000 copies within the first three days. After that time, the levels remained stable (Fig 6A). After introducing the pathogenic m.3177 G > A mutation into oocytes of *in vitro* cultured secondary follicles, we found a decline in mutant copy numbers, accompanied by an increase in WT copy numbers over the seven-day culture period (Fig 6B). To determine whether the decrease in mutant copies resulted from mtDNA degradation or mitophagy, we performed transmission electron microscopy (TEM) on the oocytes. Compared with the normal mitochondria in unedited oocytes, the mitochondria in edited oocytes appeared swollen, and an increased number of lysosomes were observed (Fig 6E-6G), suggesting activation of mitophagy. However, more direct evidence, such as mitophagy gene knockout experiments, is required to draw a confirmatory conclusion. Thus, the observed reduction in pathogenic mutations was likely due to both the elimination of mitochondria containing high levels of mutant copies and compensatory replication of WT copies.

Further analysis of digital PCR data from both *in vitro* (Fig 6C) and *in vivo* (Fig 6D) models revealed that both mutant and WT oocytes gained comparable amounts of WT mtDNA during folliculogenesis. On Day 7 of culture, WT mtDNA copy numbers in *in vitro* cultured edited oocytes had reached levels similar to those in unedited oocytes, with total mtDNA copy numbers in edited oocytes exceeding those in unedited oocytes (Fig 6C). To complement the in vitro data, we also isolated and analyzed *in vivo* matured oocytes from WT and m.3177G > A mutant mice pretreated with gonadotropins (Fig 6D). Similarly, WT mtDNA copy numbers *in* mature oocytes from mutant mice were comparable to those from WT mice (Fig 6D), although the *mutant* oocytes exhibited a wider range of heteroplasmy and total mtDNA copy numbers. Thus, during early folliculogenesis, oocytes from unedited follicles showed robust replication of WT mtDNA, whereas those from edited follicles showed both degradation of mutant mtDNA and replication of WT copies to achieve comparable total mtDNA copy numbers (Fig 6H).

## Discussion

Mitochondria are the energy factories of cells and possess their own genome- mtDNA [1]. Pathogenic mutations in mtDNA can lead to severe diseases affecting multiple tissues, including the heart, brain, and muscles. Therefore, one of the key issues in maintaining human health is preventing the spread and accumulation of these pathogenic mutations across generations [5,6].

Muller’s ratchet, first proposed by American geneticist Hermann Joseph Muller, suggests that in asexual reproduction without chromosome recombination (such as strict maternal inheritance of mtDNA [2]), mutations will accumulate continuously in the offspring, eventually leading to the extinction of the population [3,4]. It is widely accepted that the transgenerational mitochondria DNA mutations are randomly assorted based on the “bottleneck hypothesis” [45,46].

However, studies in various species, including humans [14–17], mice [7,8], Drosophila *melanogaste*r[9–12], and nematodes [13], have shown that pathogenic mutations can be eliminated during trans-generational transmission through a process known as purifying selection. Despite these findings, the nature of genetic drift remain poorly understood. Here, we provide a mechanism for the purifying selection of mitochondrial mutant DNAs during follicle development, beyond the random process mediated by the “bottleneck”.

By analyzing the heteroplasmy of oocytes at different follicle stages in the mouse ovary and observing mtDNA dynamics in secondary follicles cultured to antral follicles *in vitro*, we provide evidence that purifying selection acts during folliculogenesis. Although the extent of heteroplasmy decreases in vitro is more pronounced than those in vivo, the general trend of decreasing heteroplasmy is apparent, and the observed quantitative differences could be due to different starting points (tail biopsy for in vivo data and 24h time point for in vitro data) of individual mouse and difficulties involved in in vivo evaluation of heteroplasmy. Further experiments using more mutation models are needed to extend the present findings.

From primordial germ cells (PGCs) to live pups, germ cells undergo a long process, including follicular development, oocyte maturation, fertilization, and pre- and post-implantation embryo development. It holds important significance that purifying selection acts during folliculogenesis rather than other stages. First, unlike in somatic cells, mtDNA in oocytes undergoes massive amplification during folliculogenesis, allowing purifying selection to compensate for the lack of DNA repair mechanisms and prevent mutation accumulation. Second, selection during follicle development minimize mitochondrial dysfunction before the critical stage of oocyte maturation, thereby ensuring optimal embryo development and reduces the risk of adverse events like stillbirths.

However, it is important to emphasize that different views exist in this field. In Mitalipov’s lab, Ma et al implied that purifying selection does not act in the maternal germline per se, but during post-implantation development [47]. The reasons behind this discrepancy warrant further investigation. In our view, this difference may stem from the distinct models used in the two studies. The animals in their study harbored multiple mtDNA mutations caused by PolG deficiency, rather than a single pathogenic mutation. As is known, not all pathogenic mtDNA mutations undergo negative selection. A well-known phenomenon of positive selection exists for certain mutations, such as MELAS-associated m.3243A > G mutation [48] or other currently unknown sites. Therefore, the presence of multiple mtDNA mutations in a single model may complicate the interpretation of purifying selection, thus explaining the difference between these two works. One possible explanation for why some pathogenic mtDNA mutations escape purifying selection is that their functional impact may be modulated by the nuclear genetic background or by cellular compensatory mechanisms throughout the reproductive lifespan, thereby reducing their apparent pathogenicity to a level insufficient to elicit purifying selection.

Beyond the timing of purifying selection, the level at which it occurs is also a critical issue [44,49]. To reduce pathogenic mutations, purifying selection could act at four levels: the genomic level (via direct degradation or repair of mutant mtDNA), the organelle level (through mitophagy and/or preferential replication), the cellular level (via follicular atresia or apoptosis of oocytes with high heteroplasmy), and the organism level (through lethality of pups with high heteroplasmy). First, selection is unlikely to act at the genomic level, as evidenced by the unbiased transmission of synonymous mutations. Second, the increased ratio of swollen mitochondria in lysosomes found under TEM indicates that selection could occur at the mitochondrial (organelle) level. Third, purifying selection is unlikely at the cellular level, given that no follicular atresia has been found during early folliculogenesis until the early antral stage *in vivo* [50] and the survival rate of edited oocytes during folliculogenesis approaches 100%. Lastly, the unaffected litter sizes in mice carrying the m.3177 G > A mutation excluded selection at the organism level. Thus, unlike purifying selection acting on nuclear genome mutations at the organism level, the purifying selection of pathogenic mutations in the mitochondrial genome observed here most likely occurs at the organelle level during early folliculogenesis, mediated by mitochondrial demise. While we cannot completely rule out the possibility that oocytes carrying high mutation loads might be preferentially eliminated via advanced follicular atresia during early folliculogenesis *in vivo*, this hypothesis requires further investigation.

Exploring the underlying mechanism of purifying selection could also pave the way to novel clinical therapies. Mitochondrial replacement therapy (MRT) is an important method for preventing the transmission of pathogenic mtDNA mutations to offspring, but it faces technical difficulties and ethical concerns regarding the ‘tri-parental’ construct [51]. Developing small-molecule drugs to eliminate pathogenic mutations before ovulation could offer novel reproductive options for women with mtDNA diseases, bypassing ethical concerns associated with MRT. Our study pointed out two pathways involved in purifying selection: the selective elimination of mutant copies, probably by mitophagy and the preferential replication of wild-type copies, ultimately obtaining an optimal number of wild-type copies. This replication bias increases the likelihood of wild-type mitochondria been inherited to the next generation. A study in Drosophila *melanogaste*r also suggested inhibition of mutant mitochondrial replication through suppression of local translation [52]. Supporting the notion of mitophagy-mediated elimination of mutant copies, previous studies have implicated several mitophagy-related genes in this process, including *Bcl2l13*, *ulk1*, *ulk2,* and *bnip3* [9,53]. However, a study on mice carrying m.28020 G > A mutation argued against mitophagy as a mechanism of purifying selection, because there was no increased expression of mitophagy-related genes and no observable rise in lysosome in mutant oocytes compared to wildtype oocytes [54]. Boudoures et al (2017) also reported that mouse oocytes do not activate mitophagy in response to mitochondrial damage induced by diet or toxins [50]. In our study, purifying selection most likely occurs at the organelle level, as suggested by the increased lysosome observed by TEM. However, more direct evidence is needed to conclusively establish the role of mitophagy in purifying selection in mammals.

In this study, we used DdCBE to induce mtDNA mutations in cultured follicles to monitor the dynamics of heteroplasmy during folliculogenesis. However, we cannot exclude the possibility that the observed decrease in heteroplasmy of DdCBE-induced mutations is affected by the “repair” of the C > U based edits, as PCR polymerase cannot distinguish between uracil (U) and thymine (T). Future in vivo studies—along with more refined assays—will be necessary to conclusively distinguish between intermediate repair events and stable C > T edits.

Taken together, this study demonstrates the transgenerational elimination of pathogenic mutations during folliculogenesis through the degradation of mutant copies together with compensatory replication of wildtype copies. These findings advance our understanding of mitochondrial diseases and pave the way for novel therapeutic strategies.

## Supporting information

S1 Fig Effect of maternal age on purifying selection of m.3177G > A mutation. (A) The effect of maternal age on mother-pup heteroplasmy shift. The heteroplasmy shift is calculated as pup*(100%-mother)/mother/(100%-pup). Every point represents the average heteroplasmy shift of all pups in a delivery. (B) The effect of maternal age on heteroplasmy shift of growing oocytes. The heteroplasmy shift is calculated as oocyte*(100%-mother)/mother/(100%-oocyte). Every point represents the average heteroplasmy shift of all oocytes from each mouse. (C) The effect of maternal age on heteroplasmy shift of different tissues. The heteroplasmy shift is calculated as tissues heteroplasmy/ 4-week tail heteroplasmy.
(TIF)

S2 Fig Frequencies of on- and off-targeting C· G to T·A conversion along the whole-mtDNA genome of embryos and oocytes injected with 3177 or 12918 DdCBE mRNA.
(TIFF)

S1 Table TALE array binding sequence. (DOCX)

S2 Table Primers used for sanger sequence, next generation sequence, digital PCR and long-range PCR. (DOCX)

S3 Table On-target and Off-Target of mutant mice among different generations. (XLSX)

S1 Datasheet Numerical data underlying all of the graphs. (XLSX)
