# Supplementary figures and images for: Trans-generational maintenance of mitochondrial DNA integrity in oocytes during early folliculogenesis
Source: PLoS Genet. 2025 Dec 3;21(12):e1011562. doi: 10.1371/journal.pgen.1011562 (PMC12694818; doi:10.1371/journal.pgen.1011562)

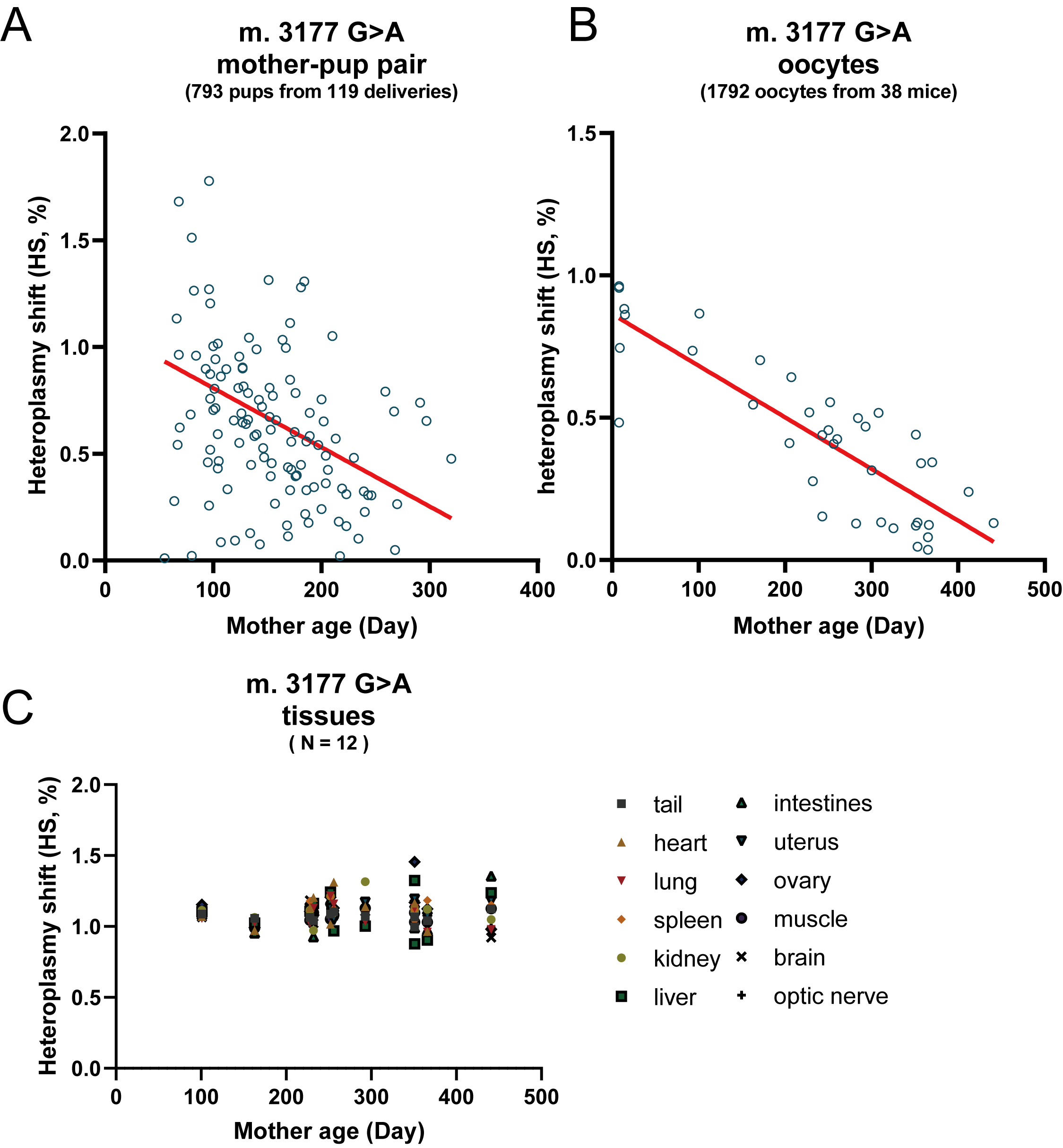

Supplement: S1 Fig — (A) The effect of maternal age on mother-pup heteroplasmy shift. The heteroplasmy shift is calculated as pup*(100%-mother)/mother/(100%-pup). Every point represents the average heteroplasmy shift of all pups in a delivery. (B) The effect of maternal age on heteroplasmy shift of growing oocytes. The heteroplasmy shift is calculated as oocyte*(100%-mother)/mother/(100%-oocyte). Every point represents the average heteroplasmy shift of all oocytes from each mouse. (C) The effect of maternal age on heteroplasmy shift of different tissues. The heteroplasmy shift is calculated as tissues heteroplasmy/ 4-week tail heteroplasmy. (TIF) [file pgen.1011562.s001.tif]
