# Supplementary material for: Trans-generational maintenance of mitochondrial DNA integrity in oocytes during early folliculogenesis
Source: PLoS Genet. 2025 Dec 3;21(12):e1011562. doi: 10.1371/journal.pgen.1011562 (PMC12694818; doi:10.1371/journal.pgen.1011562)
Supplement: S1 Table — (DOCX) [file pgen.1011562.s003.docx]

**Table S1.** TALE array binding sequence.

| Gene | Site | DdCBE pair | TALE array binding sequence |
| --- | --- | --- | --- |
| Nd1 | G3177A | L:1397C  R:1397N | 5’-TAGCCCAAACAATTTCATATGAAGTAACCATAGCTATTATCCTTTTA-3’  3’-ATCGGGTTTGTTAAAGTATACTTCATTGGTATCGATAATAGGAAAAT-5’ |
| Nd5 | G12918A | L:1397N  R:1397C | 5’-TTCCTAACAGGGTTCTACTCAAAAGACCTAATTATTGAAGCAATTAATACCTGCAA-3’  3’-AAGGATTGTCCCAAGATGAGTTTTCTGGATTAATAACTTCGTTAATTATGGACGTT-3’ |
| Nd1 | C3003T | L:1397N  R:1397C | 5’-TACCCTATCACTCACACTAGCATTAAGTCTATGAGTTCCCCTACCAATA-3’  3’-ATGGGATAGTGAGTGTGATCGTAATTCAGATACTCAAGGGGATGGTTAT-5’ |
| Nd1 | C3527T | L:1397N  R:1397C | 5’-TTTACCAGAACTCTACTCAACTAACTTCATAATAGAAGCTCTACTACTATCA-3’  3’-AAATGGTCTTGAGATGAGTTGATTGAAGTATTATCTTCGAGATGATGATAGT-5’ |
| Cox3 | C8697T | L:1397C  R:1397N | 5’-TTTCAGCCCTCCTTCTAACATCAGGTCTAGTAATATGATTTCACTATA-3’  3’-AAAGTCGGGAGGAAGATTGTAGTCCAGATCATTATACTAAAGTGATAT-5’ |
| Nd5 | G12686A | L:1397C  R:1397N | 5’-TCAAGCCAACTAGGCCTGATAATAGTGACGCTAGGAATAAACCAACCACACCTA-3’  3’-AGTTCGGTTGATCCGGACTATTATCACTGCGATCCTTATTTGGTTGGTGTGGAT-5’ |

The target base is indicated in red and binding sequence is shown in green.
