# Supplementary material for: Trans-generational maintenance of mitochondrial DNA integrity in oocytes during early folliculogenesis
Source: PLoS Genet. 2025 Dec 3;21(12):e1011562. doi: 10.1371/journal.pgen.1011562 (PMC12694818; doi:10.1371/journal.pgen.1011562)
Supplement: S2 Table — (DOCX) [file pgen.1011562.s004.docx]

**Table S2.** Primers used for sanger sequence, next generation sequence, digital PCR and long-range PCR.

|  | Primer name | Sequence (5’-3’) |
| --- | --- | --- |
| Primers for next generation sequence | 3177_BF | TCGTCGGCAGCGTCAGATGTGTATAAGAGACAG*NNNNNNNN*CTAGCAGAAACAAACCGGGC |
|  | 3177_BR | GTCTCGTGGGCTCGGAGATGTGTATAAGAGACAG*NNNNNNNN*GTCAGGCTGGCAGAAGTAATCA |
|  | 12918_BF | TCGTCGGCAGCGTCAGATGTGTATAAGAGACAG*NNNNNNNN*GACGAACAAGACATCCGAAA |
|  | 12918_BR | GTCTCGTGGGCTCGGAGATGTGTATAAGAGACAG*NNNNNNNN*GCTGTTATAGAAGTGGCGATTA |
|  | 3003_BF | TCGTCGGCAGCGTCAGATGTGTATAAGAGACAG*NNNNNNNN*GAGGTTCAAATCCTCTCCCTA |
|  | 3003_BR | GTCTCGTGGGCTCGGAGATGTGTATAAGAGACAG*NNNNNNNN*GGCTAGATGTTGC |
|  | 3527_BF | TCGTCGGCAGCGTCAGATGTGTATAAGAGACAG*NNNNNNNN*CTAGCAGAAACAAACCGGGC |
|  | 3527_BR | GTCTCGTGGGCTCGGAGATGTGTATAAGAGACAG*NNNNNNNN*GCTCGGATCCATAGGAATGTTG |
|  | 8697_BF | TCGTCGGCAGCGTCAGATGTGTATAAGAGACAG*NNNNNNNN*ACCCTCCTAGTAAGCCTATA |
|  | 8697_BR | GTCTCGTGGGCTCGGAGATGTGTATAAGAGACAG*NNNNNNNN*TTACGTCTCGTC |
|  | 12686_BF | TCGTCGGCAGCGTCAGATGTGTATAAGAGACAG*NNNNNNNN*CAACTATACTTTGCCTCGGAGC |
|  | 12686_BR | GTCTCGTGGGCTCGGAGATGTGTATAAGAGACAG*NNNNNNNN*GTCTTGTTCGTCTG |
| Primers and probe for digital PCR | Forward_Primer | CTCACTATTCGGAGCTTTACG |
|  | Reverse_Primer | GTTTGTAGAGAGTAGGATCCATTTA |
|  | WT_Probe: | /5HEX/CA+TA+T+G+AAG+TAA+CCAT/3IABkFQ/ |
|  | MUT_Probe: | /56-FAM/TA+T+A+AAGTAA+CCA+T+AGCT/3IABkFQ/ |
| Primers for long-range PCR | LR_A_F | CCAACACCGGAATGCCTAAAG |
|  | LR_A_R | CGTCGGTTTGTCATAGAAGTGT |
|  | LR_B_F | CCATCTTAGTTTTCGCAGCC |
|  | LR_B_R | CCTCTTCACTGAAAGGTCAATTTCAC |

**N* represented barcode.
